# The genome sequence of the common sole, *Solea solea *(Linnaeus, 1758)

**DOI:** 10.12688/wellcomeopenres.23353.1

**Published:** 2025-01-20

**Authors:** Enora Geslain, Filip A.M. Volckaert, Ann M. Mc Cartney, Giulio Formenti, Alice Mouton, Wellcome Sanger Institute Tree of Life Management, Samples and Laboratory team, Wellcome Sanger Institute Scientific Operations: Sequencing Operations, Wellcome Sanger Institute Tree of Life Core Informatics team, Tree of Life Core Informatics collective

**Affiliations:** 1 Laboratory of Biodiversity and Evolutionary Genomics, KU Leuven, Leuven, Belgium; 2 Genomics Institute, University of California Santa Cruz, Santa Cruz, California, USA; 3 The Vertebrate Genome Laboratory, The Rockefeller University, New York, New York, USA; 4 Department of Biology, University of Florence, Sesto Fiorentino, Italy; 5 InBios-Conservation Genetics Laboratory, University of Liege, Liege, Belgium; 6 Leibniz Institut für Zoo und Wildtierforschung, Berlin, Germany

**Keywords:** Solea solea, common sole, genome sequence, chromosomal, Pleuronectiformes

## Abstract

We present a genome assembly from an individual female *Solea solea* (Linnaeus, 1758) (the common sole; Chordata; Actinopteri; Pleuronectiformes; Soleidae). The genome sequence spans 643.80 megabases. Most of the assembly (97.81%) is scaffolded into 21 chromosomal pseudomolecules. The mitochondrial genome has also been assembled and is 17.03 kilobases in length. Gene annotation of this assembly on Ensembl identified 21,646 protein-coding genes.

## Species taxonomy

Eukaryota; Opisthokonta; Metazoa; Eumetazoa; Bilateria; Deuterostomia; Chordata; Craniata; Vertebrata; Gnathostomata; Teleostomi; Euteleostomi; Actinopterygii; Actinopteri; Neopterygii; Teleostei; Osteoglossocephalai; Clupeocephala; Euteleosteomorpha; Neoteleostei; Eurypterygia; Ctenosquamata; Acanthomorphata; Euacanthomorphacea; Percomorphaceae; Carangaria; Pleuronectiformes; Pleuronectoidei; Soleidae; *Solea*; *Solea solea*, (Linnaeus, 1758) (NCBI:txid90069).

## Background

The demersal flatfish *Solea solea* (Linnaeus, 1758), known commonly as common sole, typically burrows in sandy and muddy bottoms at depths of less than 150 m. This species is widespread in warm and cold temperate seas, including East Atlantic continental shelf waters from Trondheim Fjord (65° N; Norway) southward, the Mediterranean, including the Sea of Marmara, Bosporus and southwestern Black Sea, and the northwestern African coastal waters southward to Senegal, including Cape Verde (5° N) ( Froese & Pauly, 2024). Common sole reach sizes of max. 70 cm, but more commonly 15 to 45 cm and may reach 40 years of age ( ICES, 2006). Planktonic larvae feed on copepod nauplii; juveniles and adults feed nocturnally on benthic invertebrates such as polychaetes, siphons of bivalves and small crustaceans (amphipods) and young stages of echinoderms ( ICES, 2006). Common sole spawn along the coast in proximity of estuaries; postlarvae settle on inshore nursery grounds where they grow to subadults during two to three years. Recruitment is highly variable.

Common sole is a high value consumption flatfish with a well-known biology and limited genomic resources ( Gibson *et al.*, 2014). It is targeted by commercial fisheries using beam trawling, formerly electrotrawling, and to a lesser extent gill netting ( ICES, 2006; Rijnsdorp *et al.*, 2024). Major fished stocks are managed regionally by ICES without evidence for a mismatch with genetic structure ( Diopere *et al.*, 2018). Total landings of 87,120 metric tonnes were estimated in 1992 down to 31,030 metric tonnes in 2019 ( Pauly *et al.*, 2020). The International Union for the Conservation of Nature has listed common sole as “Data Deficient” because of the poor availability of data over sections of the species range ( Tous *et al.*, 2015).

Here we present, to our knowledge, the first complete chromosomal-level genome sequence reported for *Solea solea*, based on a female specimen from the Kwintebank (51.283 N; 2.65 E), North Sea, Belgium, kept at the Belgian Institute of Natural Sciences, Brussels, Belgium (voucher numbers KBIN/IRSNB/RBINS 27235 and KBIN/IRSNB/RBINS AB42614133).

## Genome sequence report

**Figure 1.**
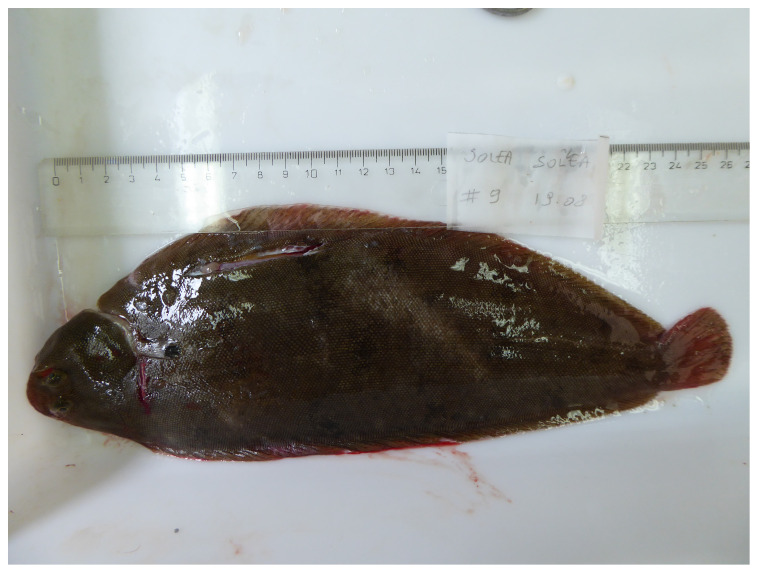
Photograph of the adult female *Solea solea* (fSolSol10) specimen used for genome sequencing.

The genome of an adult female *Solea solea* ( Figure 1) was sequenced using Pacific Biosciences single-molecule HiFi long reads, generating a total of 20.96 Gb (gigabases) from 2.27 million reads, providing approximately 32-fold coverage. Primary assembly contigs were scaffolded with chromosome conformation Hi-C data, which produced 130.51 Gb from 864.29 million reads, yielding an approximate coverage of 203-fold. Specimen and sequencing information is summarised in Table 1.

**Table 1.** Specimen and sequencing data for *Solea solea*.

Project information			
**Study title**	Solea solea (common sole)		
**Umbrella BioProject**	PRJEB61337		
**Species**	*Solea solea*		
**BioSample**	SAMEA10984647		
**NCBI taxonomy ID**	90069		
Specimen information			
**Technology**	**ToLID**	**BioSample accession**	**Organism part**
**PacBio long read sequencing**	fSolSol10	SAMEA10984681	gonad
**Hi-C sequencing**	fSolSol10	SAMEA10984680	gill animal
**RNA sequencing**	fSolSol7	SAMEA10984669	gonad
Sequencing information			
**Platform**	**Run accession**	**Read count**	**Base count (Gb)**
**Hi-C Illumina NovaSeq 6000**	ERR11242545	8.64e+08	130.51
**PacBio Sequel IIe**	ERR11242127	2.27e+06	20.96
**RNA Illumina NovaSeq 6000**	ERR12245556	7.92e+07	11.96
**RNA Illumina NovaSeq 6000**	ERR11242544	7.51e+07	11.34

Assembly errors were corrected during manual curation: including 22 missing joins or mis-joins and three haplotypic duplications. This reduced the scaffold number by 2.8% and increased the scaffold N50 by 0.21%. The final assembly has a total length of 643.80 Mb in 242 sequence scaffolds with a scaffold N50 of 29.1 Mb ( Table 2). The total count of gaps in the scaffolds is 373.

**Table 2.** Genome assembly data for *Solea solea*, fSolSol10.1.

Genome assembly		
Assembly name	fSolSol10.1	
Assembly accession	GCA_958295425.1	
*Accession of alternate haplotype*	*GCA_958295035.1*	
Span (Mb)	643.80	
Number of contigs	616	
Number of scaffolds	242	
Longest scaffold (Mb)	47.06	
Assembly metrics *		*Benchmark*
Contig N50 length (Mb)	2.6	*≥ 1 Mb*
Scaffold N50 length (Mb)	29.1	*= chromosome N50*
Consensus quality (QV)	60.9	*≥ 40*
*k*-mer completeness	99.36% (combined assemblies)	*≥ 95%*
BUSCO v 5.3.2 lineage: actinopterygii_odb10 **	C:98.3%[S:97.2%,D:1.1%], F:0.5%,M:1.2%,n:3,640	*S > 90%* *D < 5%*
Percentage of assembly mapped to chromosomes	97.81%	*≥ 90%*
Sex chromosomes	Not identified	*localised homologous pairs*
Organelles	Mitochondrial genome: 17.03 kb	*complete single alleles*
Genome annotation of assembly GCA_958295425.1 at Ensembl		
Number of protein-coding genes	21,646	
Number of non-coding genes	1,553	
Number of gene transcripts	51,064	

* Assembly metric benchmarks are adapted from Rhie *et al.* (2021) and the Earth BioGenome Project Report on Assembly Standards September 2024. ** A full set of BUSCO scores is available at https://blobtoolkit.genomehubs.org/view/fSolSol10_1/dataset/fSolSol10_1/busco.

**Figure 2.**
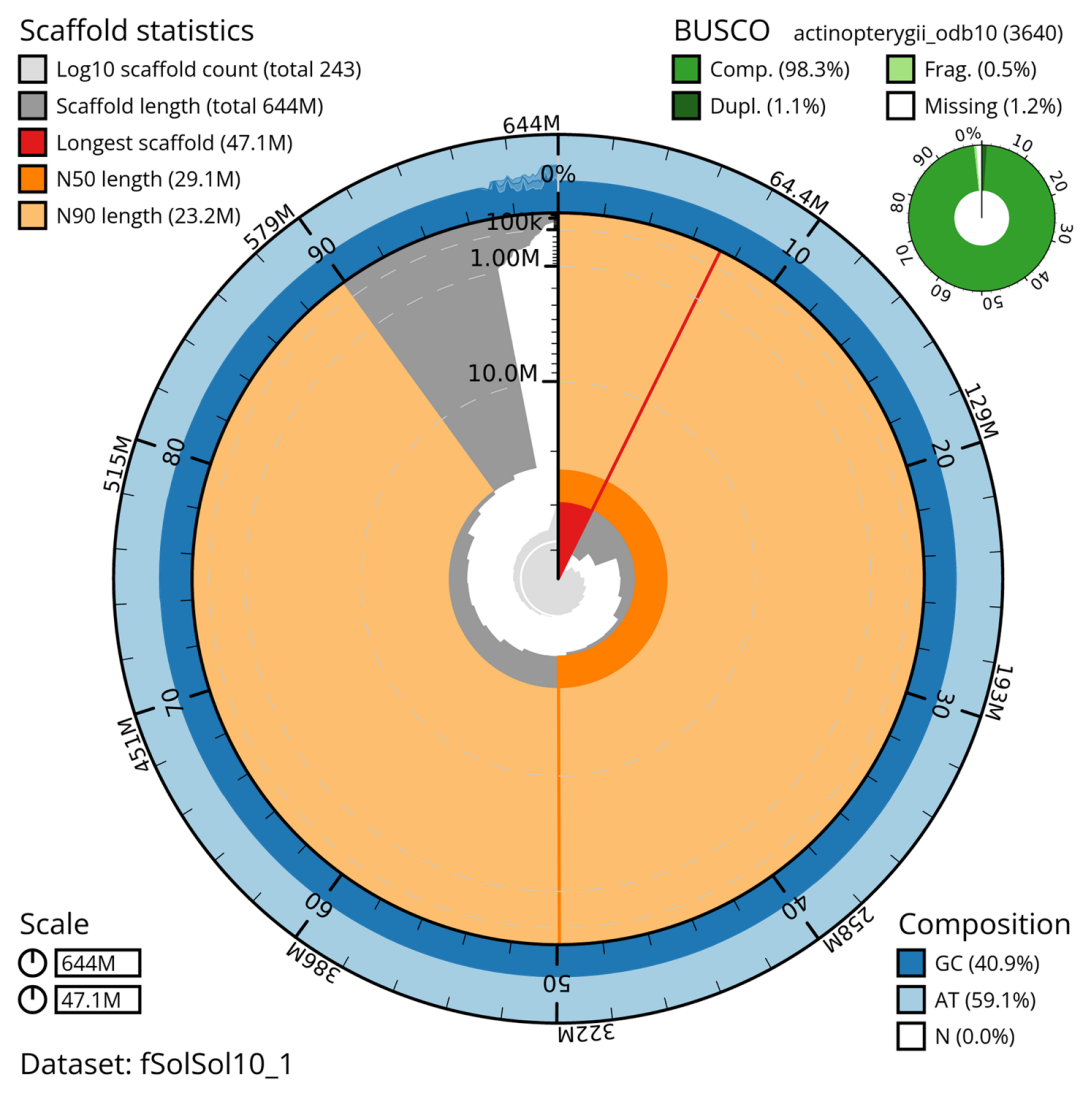
Genome assembly of *Solea solea*, fSolSol10.1: metrics. The BlobToolKit snail plot shows N50 metrics and BUSCO gene completeness. The main plot is divided into 1,000 bins around the circumference with each bin representing 0.1% of the 643,774,986 bp assembly. The distribution of scaffold lengths is shown in dark grey with the plot radius scaled to the longest scaffold present in the assembly (47,064,963 bp, shown in red). Orange and pale-orange arcs show the N50 and N90 scaffold lengths (29,068,826 and 23,155,029 bp), respectively. The pale grey spiral shows the cumulative scaffold count on a log scale with white scale lines showing successive orders of magnitude. The blue and pale-blue area around the outside of the plot shows the distribution of GC, AT and N percentages in the same bins as the inner plot. A summary of complete, fragmented, duplicated and missing BUSCO genes in the actinopterygii_odb10 set is shown in the top right. An interactive version of this figure is available at https://blobtoolkit.genomehubs.org/view/fSolSol10_1/dataset/fSolSol10_1/snail.

**Figure 3.**
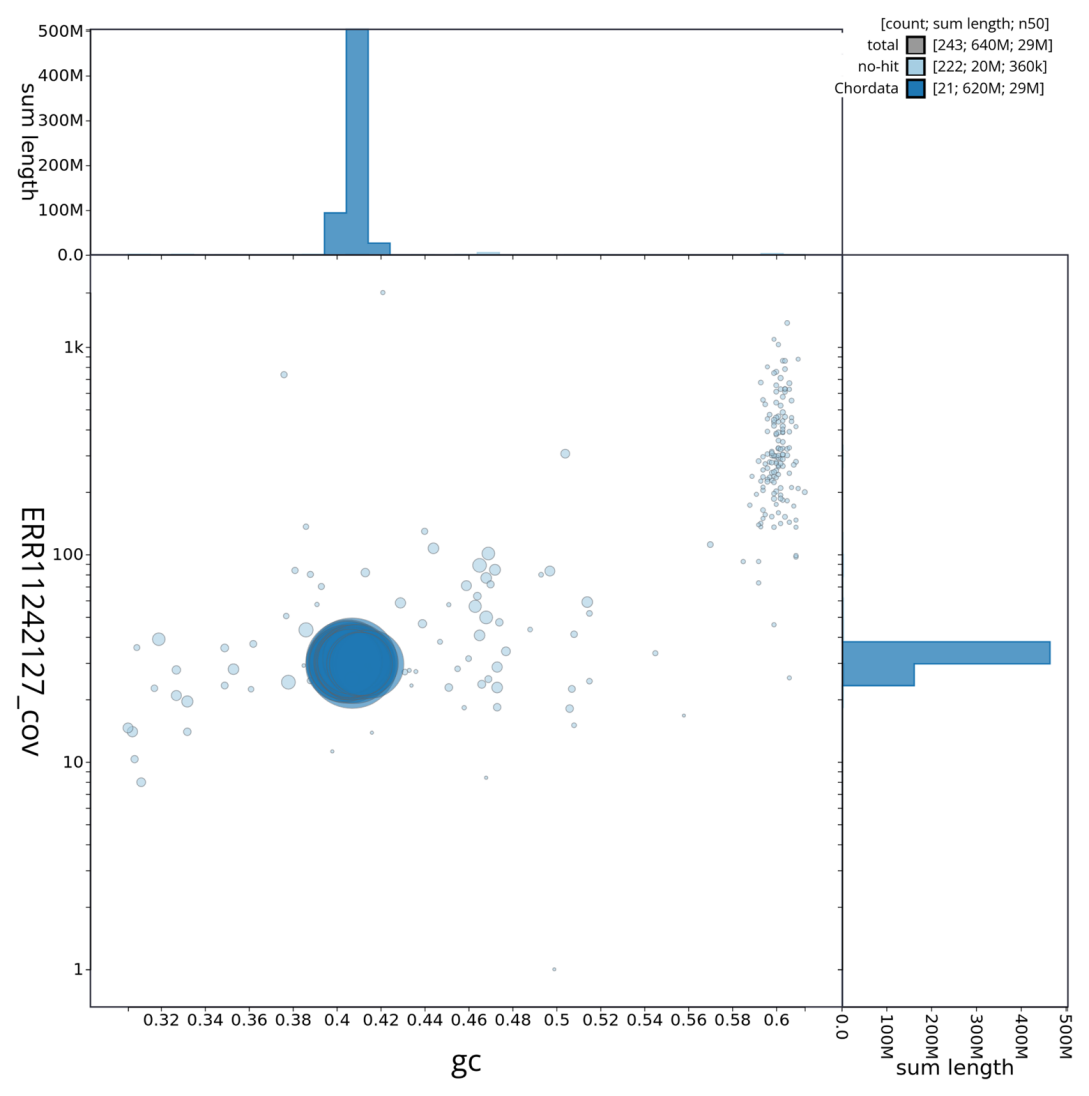
Genome assembly of *Solea solea*, fSolSol10.1: Blob plot of base coverage in ERR11242127 against GC proportion for sequences in assembly fSolSol10.1. Sequences are coloured by phylum. Circles are sized in proportion to sequence length. Histograms show the distribution of sequence length sum along each axis. An interactive version of this figure is available at https://blobtoolkit.genomehubs.org/view/fSolSol10_1/dataset/fSolSol10_1/blob.

**Figure 4.**
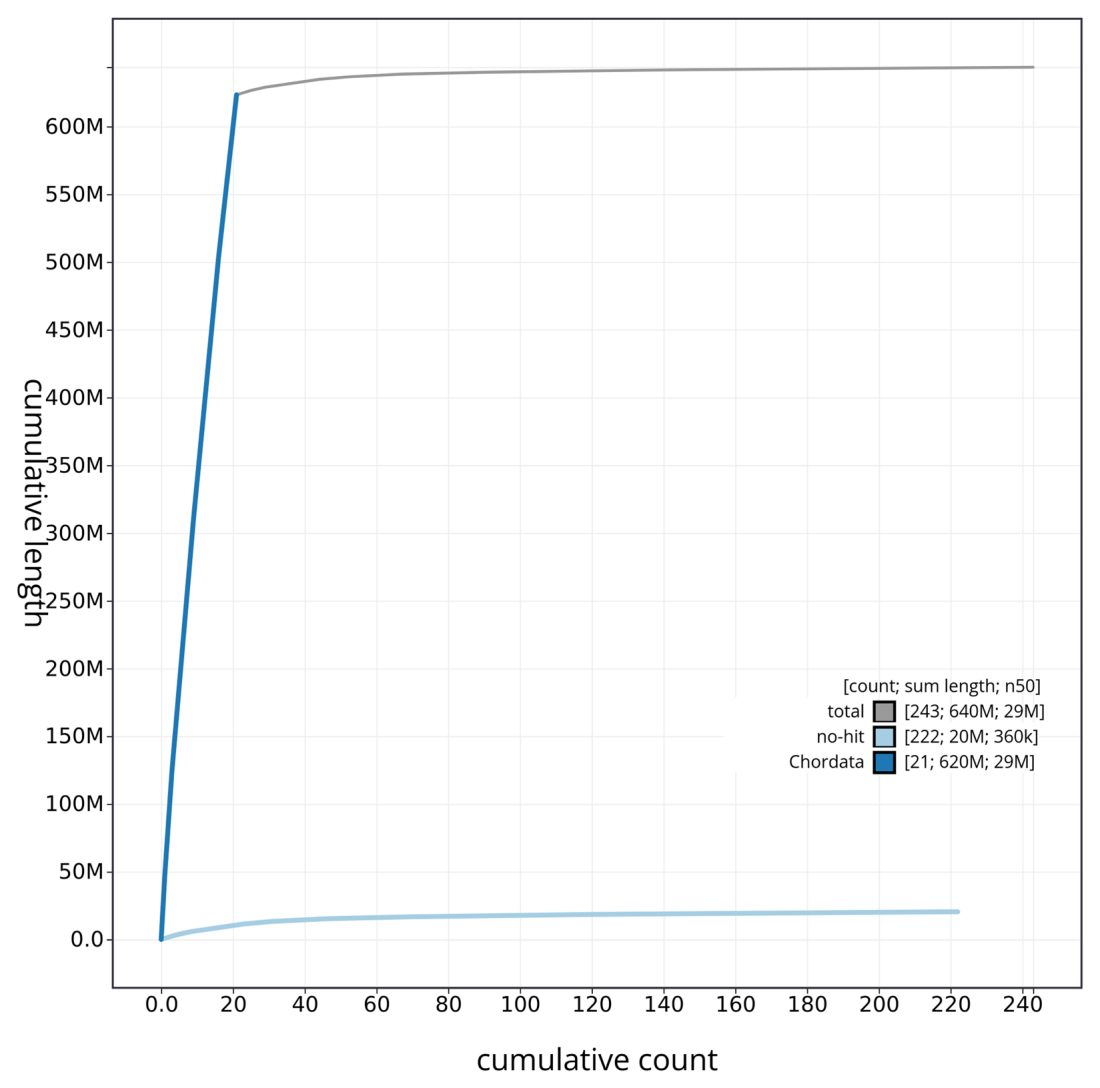
Genome assembly of *Solea solea* fSolSol10.1: BlobToolKit cumulative sequence plot. The grey line shows cumulative length for all sequences. Coloured lines show cumulative lengths of sequences assigned to each phylum using the buscogenes taxrule. An interactive version of this figure is available at https://blobtoolkit.genomehubs.org/view/fSolSol10_1/dataset/fSolSol10_1/cumulative.

The snail plot in Figure 2 provides a summary of the assembly statistics, indicating the distribution of scaffold lengths and other assembly metrics. Figure 3 shows the distribution of scaffolds by GC proportion and coverage. Figure 4 presents a cumulative assembly plot, with separate curves representing different scaffold subsets assigned to various phyla, illustrating the completeness of the assembly.

**Figure 5.**
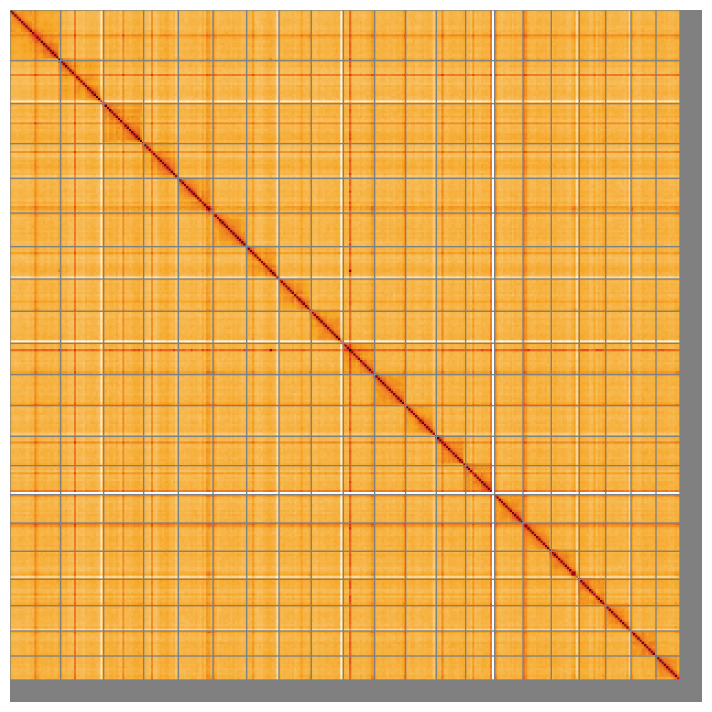
Genome assembly of *Solea solea* fSolSol10.1: Hi-C contact map of the fSolSol10.1 assembly, visualised using HiGlass. Chromosomes are shown in order of size from left to right and top to bottom. An interactive version of this figure may be viewed at https://genome-note-higlass.tol.sanger.ac.uk/l/?d=FSUlLwVQRfiQQBsuU_Y3AQ.

Most of the assembly sequence (97.81%) was assigned to 21 chromosomal-level scaffolds. Chromosome-scale scaffolds confirmed by the Hi-C data are named in order of size ( Figure 5; Table 3). While not fully phased, the assembly deposited is of one haplotype. Contigs corresponding to the second haplotype have also been deposited. The mitochondrial genome was also assembled and can be found as a contig within the multifasta file of the genome submission.

**Table 3.** Chromosomal pseudomolecules in the genome assembly of *Solea solea*, fSolSol10.

INSDC accession	Name	Length (Mb)	GC%
OY282534.1	1	47.06	40.5
OY282535.1	2	40.01	40.5
OY282536.1	3	37.3	41.0
OY282537.1	4	26.89	41.0
OY282538.1	5	32.35	40.5
OY282539.1	6	32.19	40.5
OY282540.1	7	31.38	40.5
OY282541.1	8	30.09	40.5
OY282542.1	9	29.98	40.5
OY282543.1	10	29.76	40.5
OY282544.1	11	29.07	40.5
OY282545.1	12	29.01	40.5
OY282546.1	13	28.33	40.5
OY282547.1	14	27.38	40.5
OY282548.1	15	26.54	40.5
OY282549.1	16	26.39	41.5
OY282550.1	17	25.92	41.0
OY282551.1	18	24.5	41.5
OY282552.1	19	23.71	40.5
OY282553.1	20	23.16	41.0
OY282554.1	21	22.35	41.0
OY282555.1	MT	0.02	44.0

The estimated Quality Value (QV) of the final assembly is 60.9 with *k*-mer completeness of 99.36% (combined assemblies), and the assembly has a BUSCO v5.3.2 completeness of 98.3% (single = 97.2%, duplicated = 1.1%), using the actinopterygii_odb10 reference set ( *n* = 3,640). The assembly achieves the Earth BioGenome Project reference standard of 6.C.Q61, thus exceeding the minimum reference standard of 6.C.Q40. Other quality metrics are given in Table 2.

## Genome annotation report

The *Solea solea* genome assembly (GCA_958295425.1) was annotated at the European Bioinformatics Institute (EBI) on Ensembl Rapid Release. The resulting annotation includes 51,064 transcribed mRNAs from 21,646 protein-coding and 1,553 non-coding genes ( Table 2; https://rapid.ensembl.org/Solea_solea_GCA_958295425.1/Info/Index). The average transcript length is 21,373.58. There are 2.20 coding transcripts per gene and 13.64 exons per transcript.

## Methods

### Sample acquisition

An adult *Solea solea* (specimen ID ERGA_FV_BE_019, ToLID fSolSol10) was collected from Kwintebank (North Sea; latitude 51.28, longitude 2.65) on 2021-08-19. The specimen was collected, identified and preserved by Filip A.M. Volckaert (KU Leuven). This specimen was used for genome sequencing and Hi-C data for scaffolding.

The specimen used for RNA sequencing (ToLID fSolSol7) was a juvenile specimen collected from Wenduinebank (W03) (North Sea; latitude 51.28, longitude 2.95) on 2021-07-21. The specimen was collected, identified and preserved by Filip A.M. Volckaert (KU Leuven).

### Nucleic acid extraction

The workflow for high molecular weight (HMW) DNA extraction at the Wellcome Sanger Institute (WSI) Tree of Life Core Laboratory includes a sequence of core procedures: sample preparation and homogenisation, DNA extraction, fragmentation and purification. Detailed protocols are available on protocols.io ( Denton *et al.*, 2023). The fSolSol10 sample was weighed and dissected on dry ice ( Jay *et al.*, 2023) and gonad tissue was cryogenically disrupted using the Covaris cryoPREP ^®^ Automated Dry Pulverizer ( Narváez-Gómez *et al.*, 2023).

HMW DNA was extracted using the Automated MagAttract v1 protocol ( Sheerin *et al.*, 2023). DNA was sheared into an average fragment size of 12–20 kb in a Megaruptor 3 system ( Todorovic *et al.*, 2023). Sheared DNA was purified by solid-phase reversible immobilisation, using AMPure PB beads to eliminate shorter fragments and concentrate the DNA ( Strickland *et al.*, 2023). The concentration of the sheared and purified DNA was assessed using a Nanodrop spectrophotometer and Qubit Fluorometer using the Qubit dsDNA High Sensitivity Assay kit. Fragment size distribution was evaluated by running the sample on the FemtoPulse system.

RNA was extracted from gonad tissue of fSolSol7 in the Tree of Life Laboratory at the WSI using the RNA Extraction: Automated MagMax™ *mir*Vana protocol ( do Amaral *et al.*, 2023). The RNA concentration was assessed using a Nanodrop spectrophotometer and a Qubit Fluorometer using the Qubit RNA Broad-Range Assay kit. Analysis of the integrity of the RNA was done using the Agilent RNA 6000 Pico Kit and Eukaryotic Total RNA assay.

### Hi-C preparation

Tissue from the gill of the fSolSol10 sample was processed at the WSI Scientific Operations core, using the Arima-HiC v2 kit. Tissue (stored at –80 °C) was fixed, and the DNA crosslinked using a TC buffer with 22% formaldehyde. After crosslinking, the tissue was homogenised using the Diagnocine Power Masher-II and BioMasher-II tubes and pestles. Following the kit manufacturer's instructions, crosslinked DNA was digested using a restriction enzyme master mix. The 5’-overhangs were then filled in and labelled with biotinylated nucleotides and proximally ligated. An overnight incubation was carried out for enzymes to digest remaining proteins and for crosslinks to reverse. A clean up was performed with SPRIselect beads prior to library preparation.

### Library preparation and sequencing

Pacific Biosciences SMRTbell libraries were constructed using the Revio HiFi prep kit, according to the manufacturers’ instructions. DNA sequencing was performed by the Scientific Operations core at the WSI on a Pacific Biosciences Revio instrument.

For Hi-C library preparation, DNA was fragmented to a size of 400 to 600 bp using a Covaris E220 sonicator. The DNA was then enriched, barcoded, and amplified using the NEBNext Ultra II DNA Library Prep Kit following manufacturers’ instructions. The Hi-C sequencing was performed using paired-end sequencing with a read length of 150 bp on an Illumina NovaSeq 6000 instrument.

Poly(A) RNA-Seq libraries were constructed using the NEB Ultra II RNA Library Prep kit, following the manufacturer’s instructions. RNA sequencing was performed on the Illumina NovaSeq 6000 instrument.

### Genome assembly, curation and evaluation

***Assembly***

The HiFi reads were first assembled using Hifiasm ( Cheng *et al.*, 2021) with the --primary option. Haplotypic duplications were identified and removed using purge_dups ( Guan *et al.*, 2020). The Hi-C reads were mapped to the primary contigs using bwa-mem2 ( Vasimuddin *et al.*, 2019). The contigs were further scaffolded using the provided Hi-C data ( Rao *et al.*, 2014) in YaHS ( Zhou *et al.*, 2023) using the --break option. The scaffolded assemblies were evaluated using Gfastats ( Formenti *et al.*, 2022), BUSCO ( Manni *et al.*, 2021) and MERQURY.FK ( Rhie *et al.*, 2020).

The mitochondrial genome was assembled using MitoHiFi ( Uliano-Silva *et al.*, 2023), which runs MitoFinder ( Allio *et al.*, 2020) and uses these annotations to select the final mitochondrial contig and to ensure the general quality of the sequence.

***Assembly curation***

The assembly was decontaminated using the Assembly Screen for Cobionts and Contaminants (ASCC) pipeline (article in preparation). Manual curation was primarily conducted using PretextView ( Harry, 2022), with additional insights provided by JBrowse2 ( Diesh *et al.*, 2023) and HiGlass ( Kerpedjiev *et al.*, 2018). Scaffolds were visually inspected and corrected as described by Howe *et al*. (2021). Any identified contamination, missed joins, and mis-joins were corrected, and duplicate sequences were tagged and removed. The curation process is documented at https://gitlab.com/wtsi-grit/rapid-curation (article in preparation).

***Evaluation of the final assembly***

A Hi-C map for the final assembly was produced using bwa-mem2 ( Vasimuddin *et al.*, 2019) in the Cooler file format ( Abdennur & Mirny, 2020). To assess the assembly metrics, the *k*-mer completeness and QV consensus quality values were calculated in Merqury ( Rhie *et al.*, 2020). This work was done using the “sanger-tol/readmapping” ( Surana *et al.*, 2023a) and “sanger-tol/genomenote” ( Surana *et al.*, 2023b) pipelines. The genome readmapping pipelines were developed using the nf-core tooling ( Ewels *et al.*, 2020), use MultiQC ( Ewels *et al.*, 2016), and make extensive use of the Conda package manager, the Bioconda initiative ( Grüning *et al.*, 2018), the Biocontainers infrastructure ( da Veiga Leprevost *et al.*, 2017), and the Docker ( Merkel, 2014) and Singularity ( Kurtzer *et al.*, 2017) containerisation solutions. The genome was also analysed within the BlobToolKit environment ( Challis *et al.*, 2020) and BUSCO scores ( Manni *et al.*, 2021) were calculated.

Table 4 contains a list of relevant software tool versions and sources.

**Table 4.** Software tools: versions and sources.

Software tool	Version	Source
BlobToolKit	4.2.1	https://github.com/blobtoolkit/blobtoolkit
BUSCO	5.3.2	https://gitlab.com/ezlab/busco
bwa-mem2	2.2.1	https://github.com/bwa-mem2/bwa-mem2
Cooler	0.8.11	https://github.com/open2c/cooler
Gfastats	1.3.6	https://github.com/vgl-hub/gfastats
Hifiasm	0.16.1-r375	https://github.com/chhylp123/hifiasm
HiGlass	1.11.6	https://github.com/higlass/higlass
Merqury.FK	d00d98157618f4e8d1a91 90026b19b471055b22e	https://github.com/thegenemyers/MERQURY.FK
MitoHiFi	3	https://github.com/marcelauliano/MitoHiFi
PretextView	0.2	https://github.com/wtsi-hpag/PretextView
purge_dups	1.2.5	https://github.com/dfguan/purge_dups
sanger-tol/genomenote	v1.0	https://github.com/sanger-tol/genomenote
sanger-tol/readmapping	1.1.0	https://github.com/sanger-tol/readmapping/tree/1.1.0
Singularity	3.9.0	https://github.com/sylabs/singularity
YaHS	1.2a.2	https://github.com/c-zhou/yahs

### Genome annotation

The Ensembl Genebuild annotation system ( Aken *et al.*, 2016) was used to generate annotation for the *Solea solea* assembly (GCA_958295425.1) in Ensembl Rapid Release at the EBI. Annotation was created primarily through alignment of transcriptomic data to the genome, with gap filling via protein-to-genome alignments of a select set of proteins from UniProt ( UniProt Consortium, 2019).

### Wellcome Sanger Institute – Legal and Governance

The materials that have contributed to this genome note have been supplied by a Tree of Life collaborator.

The Wellcome Sanger Institute employs a process whereby due diligence is carried out proportionate to the nature of the materials themselves, and the circumstances under which they have been/are to be collected and provided for use. The purpose of this is to address and mitigate any potential legal and/or ethical implications of receipt and use of the materials as part of the research project, and to ensure that in doing so we align with best practice wherever possible.

The overarching areas of consideration are:

•   Ethical review of provenance and sourcing of the material

•   Legality of collection, transfer and use (national and international)

Each transfer of samples is undertaken according to a Research Collaboration Agreement or Material Transfer Agreement entered into by the Tree of Life collaborator, Genome Research Limited (operating as the Wellcome Sanger Institute) and in some circumstances other Tree of Life collaborators.

## Data Availability

European Nucleotide Archive: *Solea solea* (common sole). Accession number PRJEB61337; https://identifiers.org/ena.embl/PRJEB61337. The genome sequence is released openly for reuse. The *Solea solea* genome sequencing initiative is part of the European Reference Genome Atlas (ERGA) pilot project. All raw sequence data and the assembly have been deposited in INSDC databases. Raw data and assembly accession identifiers are reported in Table 1 and Table 2. Metadata for specimens, BOLD barcode results, spectra estimates, sequencing runs, contaminants and pre-curation assembly statistics are given at https://links.tol.sanger.ac.uk/species/90069.

## Author information

Members of the Wellcome Sanger Institute Tree of Life Management, Samples and Laboratory team are listed here: https://doi.org/10.5281/zenodo.12162482.

Members of Wellcome Sanger Institute Scientific Operations: Sequencing Operations are listed here: https://doi.org/10.5281/zenodo.12165051.

Members of the Wellcome Sanger Institute Tree of Life Core Informatics team are listed here: https://doi.org/10.5281/zenodo.12160324.

Members of the Tree of Life Core Informatics collective are listed here: https://doi.org/10.5281/zenodo.12205391.
