# Supplementary material for: The genome sequence of the common sole, Solea solea (Linnaeus, 1758)
Source: Wellcome Open Res. 2025 Jan 20;10:25. [Version 1] doi: 10.12688/wellcomeopenres.23353.1 (PMC11829150; doi:10.12688/wellcomeopenres.23353.1)
Supplement: Peer review history

# Reviewer report: Reviewer response for version 1

**DOI:** 10.21956/wellcomeopenres.25741.r117558

**Published:** 2025-02-14

**Authors:** Shigehiro Kuraku

**Affiliations:** 1 National Institute of Genetics, Shizuoka, Japan

The manuscript soundly reports the chromosome-scale assembly of the common sole genome. I expect it to be recognized by researchers working on the species and used for fishery science as well as cross-species comparisons etc. for biodiversity studies in general. I suggest incorporating the changes requested below before it is accepted for indexing.

 I wonder if the part 'Most of the assembly sequence (97.81%)' refers to the proportion in length or sequence number.

 Is the sex of the animal used for sequencing unknown? If known, that should be clarified in the manuscript.

 The details of the parameter choice in the Hi-C read mapping step (with bwa-mem2) should be included in the Methods. Also, wasn't the step to make duplicate reads (recommended by the YaHS developer) inserted?

Are sufficient details of methods and materials provided to allow replication by others?

Yes

Is the rationale for creating the dataset(s) clearly described?

Yes

Are the datasets clearly presented in a useable and accessible format?

Yes

Are the protocols appropriate and is the work technically sound?

Yes

Reviewer Expertise:

vertebrate evolutionary genomics, developmental biology

I confirm that I have read this submission and believe that I have an appropriate level of expertise to confirm that it is of an acceptable scientific standard.

# Reviewer report: Reviewer response for version 1

**DOI:** 10.21956/wellcomeopenres.25741.r117899

**Published:** 2025-02-10

**Authors:** Huria Marnis

**Affiliations:** 1 National Research and Innovation Agency (BRIN), Cibinong, Indonesia

The present study is an interesting work providing genome sequence of common sole. It can be useful for further studies related to genome references in flatfish and other aquatic species. However, some minor issues exist in the text. They should be addressed before the manuscript is suitable for indexing.

**Genome sequence report**

 Line 1 – use a common name to mention the species. The scientific name is only included at the first appearance of species in the text. Apply it thorough manuscript!

**Methods**

*Sample acquisition*

 First paragraph, In the abstract, the authors mention that authors have chosen a single female specimen, but it should state in the methods and explain why a single female specimen was chosen for genome sequencing.

 First paragraph, last sentence:

 What were tissue samples collected?

*Was the specimen freshly caught or stored before processing?*

*Was it preserved immediately after collection to prevent degradation?*

 Was it from a wild population or aquaculture setting? Mention it!

*Add details on the handling time from capture to preservation to ensure DNA/RNA integrity.*

 First paragraph and second paragraph, The RNA-seq sample came from a different specimen (juvenile instead of adult female). Was this intentional? If so, how might developmental stage differences impact gene annotation?

*Nucleic acid extraction*

 Mention DNA quality assessment (e.g., purity ratios, integrity checks).

*Library Preparation and Sequencing*

 Were additional quality control steps performed?

Are sufficient details of methods and materials provided to allow replication by others?

Yes

Is the rationale for creating the dataset(s) clearly described?

Yes

Are the datasets clearly presented in a useable and accessible format?

Yes

Are the protocols appropriate and is the work technically sound?

Yes

Reviewer Expertise:

molecular genetic

I confirm that I have read this submission and believe that I have an appropriate level of expertise to confirm that it is of an acceptable scientific standard.
